# Cost-effective sol-gel synthesis of porous CuO nanoparticle aggregates with tunable specific surface area

**DOI:** 10.1038/s41598-019-48020-8

**Published:** 2019-08-13

**Authors:** Lars Dörner, Claudia Cancellieri, Bastian Rheingans, Marc Walter, Ralf Kägi, Patrik Schmutz, Maksym V. Kovalenko, Lars P. H. Jeurgens

**Affiliations:** 1 Empa, Swiss Federal Laboratories for Materials Science and Technology, Laboratory for Joining Technologies & Corrosion, Dübendorf, Switzerland; 2 ETH Zürich, Department of Chemistry and Applied Biosciences, Zürich, Switzerland; 3 Eawag, Swiss Federal Institute of Aquatic Science and Technology, Department Process Engineering, Dübendorf, Switzerland; 4 Empa, Swiss Federal Laboratories for Materials Science and Technology, Laboratory for Thin Films and Photovoltaics, Dübendorf, Switzerland

**Keywords:** Nanoparticle synthesis, Nanoparticles, Nanoparticles

## Abstract

CuO nanoparticles (NPs) are applied in various key technologies, such as catalysis, energy conversion, printable electronics and nanojoining. In this study, an economic, green and easy-scalable sol-gel synthesis method was adopted to produce submicron-sized nanoporous CuO NP aggregates with a specific surface area > 18 m²/g. To this end, a copper-carbonate containing precursor was precipitated from a mixed solution of copper acetate and ammonia carbonate and subsequently calcinated at *T* ≥ 250 °C. The thus obtained CuO nanopowder is composed of weakly-bounded agglomerates, which are constituted of aggregated CuO NPs with a tunable size in the range of 100–140 nm. The CuO aggregates, in turn, are composed of equi-axed primary crystallites with a tunable crystallite size in the range of 20–40 nm. The size and shape of the primary CuO crystallites, as well as the nanoporosity of their fused CuO aggregates, can be tuned by controlled variation of the degree of supersaturation of the solution via the pH and the carbonate concentration. The synthesized submicron-sized CuO aggregates can be more easily and safely processed in the form of a solution, dispersion or paste than individual NPs, while still offering the same enhanced reactivity due to their nanoporous architecture.

## Introduction

Advanced nanotechnologies in the fields of catalysis, energy conversion, storage and sensing devices rely on the accurate control of the shape and the size of materials from the nano- up to the micrometer scale. This requirement has boosted the development of a wide range of synthesis techniques for producing metallic, insulating and semiconducting nanoparticles (NP) of controllable sizes and shapes (cf. reviews^1^ and^2^). Reported studies on engineered and environmental NP-based systems generally focus on the size and shape of the smallest undividable entity (or building block), commonly referred to as the primary particle (or crystallite). However, successive manufacturing and processing steps (or environmental exposure) generally induce aggregation and/or agglomeration of primary NPs into larger entities with sizes of up to several microns. NP a*ggregates* (or secondary particles) consist of strongly bonded or fused primary NPs, which cannot be separated by subsequent handling and processing steps (i.e. the aggregation process is irreversible). NP *agglomerates* comprise assemblies of more weakly bound primary NPs and NP aggregates (and/or a mixture thereof), which can be separated into their individual constituents by providing sufficient external energy and stabilized through the addition of suitable dispersion agents^3^. For practical applications, the actual properties of the nanomaterial (e.g. specific surface area, chemical reactivity, dispersibility and toxicity) will be governed by the size, shape and density (i.e. nanoporosity) of the NP aggregates and/or agglomerates and not solely by the primary particles^4,5^. For example, the extent to which the internal surface of a loosely clustered (and thus nanoporous) NP aggregate is accessible to interpenetrating gaseous or liquid species will critically influence its dispersibility, chemical reactivity and sintering behavior, which are of key importance for the development of e.g. catalysis, printed electronics and joining technologies^6,7^. Notably, sub-micron sized nanoporous NP aggregates also have the advantage that they can be more easily (i.e. both from handling and safety point of view) processed than individual nanoparticles, while still offering the same dimensionality-enabled properties and performance due to their nanoporous architecture. Hence it is crucial to evaluate and tune the existing NP synthesis routes for accurate control of size, shape and cluster density of the NP aggregates, which has been largely disregarded up to date. The present study addresses the fabrication of cupric (CuO) NP aggregates by cost-effective, environmental-friendly and scalable chemical synthesis methods with a particular focus on the controlled variation of size, shape and cluster density (nanoporosity) of the primary crystallites constituting the NP aggregates. It is demonstrated that the default synthesis route can be optimized to enlarge the specific surface area (SSA) of the resulting CuO NP aggregates and thereby enhance their reactivity and sintering ability when processed in the form of a dispersion, paste or nanocomposite^8^. Various synthesis routes for the production of monodisperse CuO NPs and micro-sized spheres have been established in the last decades, but to our knowledge, no reports concern the chemical synthesis of *nanoporous* CuO NP aggregates in the range of 100 nm.

Copper oxide finds broad applications in a wide range of different technologies, such as catalysis, energy conversion, magnetic storage, energy storage, thermites, as well as superconductors. Notably, the copper-oxygen system contains two stable stoichiometric oxides, cuprous (Cu_2_O) and cupric (CuO) oxide. Cuprous oxide has a cubic crystal structure with a direct band gap in the range of 2.0–2.5 eV, whereas cupric oxide has a monoclinic crystal structure with a lower band gap in the range of 1.3 eV–1.7 eV^9^. For our envisioned applications in the field of nanojoining^10–12^, the use of single-phase cupric CuO NPs is preferred^13,14^, since it presents the most stable Cu oxide phase according to bulk thermodynamics^15^. Over the years, a wide variety of techniques have been developed and evaluated for synthesizing single-phase CuO NPs with controllable size and shape, including nanoparticles (0D), nanowires (1D), nanoplatelets (2D), thin films (2D), as well as their respective 3D hierarchical structures; see review papers^1^ and^2^ and references therein. Commonly used CuO synthesis methods include milling^16^, electrodeposition^17^, sonochemical synthesis^18^, ultrasonic spray pyrolysis^19^ and hydrothermal synthesis^20,21^ (see also reviews^1^ and^2^). Solution-based methods, including sol-gel synthesis, have proven to be the most effective method for easy and cost-effective production of CuO NPs^22^. For example, a Cu-hydroxide precursor can be precipitated from an aging Cu(II)-salt solution, which is subsequently transformed into CuO by calcination of the extracted precipitate at elevated temperatures^23^. The size and shape of the precipitated precursor phase strongly depends on the chemical speciation of the Cu(II) cation complexes in the ageing solution, which can be tailored by variation of the type and concentration of complexing anions (e.g. SO_4_^2−^, Cl^−^, NO_3_^−^; in addition of OH^−^) and the pH^24,25^. Inorganic or organic additives, such as urea, polyethylene glycol or polyvinylpyrrolidone can be added to steer the precipitation and aggregation of the precursor phase, thus enabling the formation of specific hierarchical CuO nanostructures^22,26^. For example, Cu(NO_3_)-salt solutions in the presence of urea were aged at 90 °C to yield spherically-shaped malachite (CuCO_3_·Cu(OH)_2_) precursor particles, which after calcination at 700–800 °C transform into micro-sized spherical CuO particles^27^. Reproducible results can only be obtained if key factors such as the pH, type and concentrations of the reactants are well controlled^22^.

In the present study, a simple, green and low-cost sol-gel synthesis method based on a copper-carbonate species containing precursor phase, using copper acetate and ammonia carbonate salts without the addition of any additives, was selected and optimized to produce loosely clustered (and thus nanoporous) CuO NP aggregates with an average size in the range of 100–140 nm and a resulting SSA that is significantly higher than typical SSA values in the range of 10–15 m²/g, as reported for commercially available high-purity (i.e. ≥99.99%) CuO nanopowders. Sol-Gel processes are usually used to synthesize nonmetallic inorganic materials from particle dispersions. They are easily upscalable and can be conducted with cheap and non-toxic chemicals^28^. Importantly, the synthesis was performed without organic additives (which contaminate the product phase and complicates the complex forming process of precursor phases). The evolution of the crystal structure, size and shape of the primary crystallites, which develop from the precursor phase during the calcination treatment, were monitored by measuring the peak broadening through high temperature *in-situ* XRD. The size, shape and cluster density of the resulting CuO aggregates, as constituted of individual clusters of firmly bonded primary CuO crystallites, was investigated by complementary analytical techniques, including Scanning Electron Microscopy (SEM), Transmission Electron Microscopy (TEM), Dynamic Light Scattering (DLS) and Brunauer-Emmett-Teller (BET) analysis. Key parameters in the sol-gel synthesis procedure were identified (*e*.*g*. the supersaturation level, carbonate concentration and the resulting pH of the mixed solution) and tuned to obtain an enhanced SSA of the synthesized CuO nanopowder for the targeted applications.

## Experimental

### Material synthesis

Synthetic malachite can be prepared by reacting a solution of copper(II) nitrate with a solution containing the equivalent amount of sodium carbonate at room temperature; *i*.*e*. the copper-to-carbonate concentration ratio is fixed at [CO_3_^2−^]/[Cu^2+^] = 1.0^29^. Accordingly, solutions were prepared by mixing stoichiometric amounts of fresh aqueous 15 mM copper acetate (Sigma Aldrich) and 15 mM ammonia carbonate (Alfa Aesar) solutions at room temperature, while continuously stirring the mixed solution^30^. This resulted in a pH of 5.8 for the mixed solution, which remained stable during a stirring duration of 2 h, as monitored using a pH-meter (Metrohm 914 pH/Conductometer). Upon combining the starting solutions at room temperature (further referred to as mixed solution), precipitation of particles (the gel) from the blue-greenish solution was immediately observed. In an acidic environment, copper(II) ions would remain in solution without forming precipitates over the described time period. For more neutral environments of a pH above 5.6 (as is the case for the present study; see above), complexation of Cu^2+^ leads to the formation of precipitates. Due to the supersaturation of the solution with respect to the formation of malachite, successive precipitation of the copper(II) complexes and gel formation occurs, and the composition of this precursor phase moves towards the composition of malachite. The chemical reaction to malachite can be written as

1 $$2{{\rm{Cu}}}^{2+}+2{{\rm{CO}}}_{3}^{2-}+{{\rm{H}}}_{2}{\rm{O}}\to {{\rm{CuCO}}}_{3}\cdot {\rm{Cu}}{({\rm{OH}})}_{2}+{{\rm{CO}}}_{2.}$$

When the pH exceeds 8.5, the copper-carbonate-hydroxide precursor phase complexes would gradually dissolve over time by ammonia leaching (resp. they would not even be formed), eventually leading to the formation of primarily copper(II) tetra ammine complexes^31^, i.e.

2 $${{\rm{C}}{\rm{u}}{\rm{C}}{\rm{O}}}_{3}\cdot {\rm{C}}{\rm{u}}{({\rm{O}}{\rm{H}})}_{2}+6{{\rm{N}}{\rm{H}}}_{4}{\rm{O}}{\rm{H}}+{({{\rm{N}}{\rm{H}}}_{4})}_{2}{{\rm{C}}{\rm{O}}}_{3}\to 2{\rm{C}}{\rm{u}}{({{\rm{N}}{\rm{H}}}_{3})}_{4}{{\rm{C}}{\rm{O}}}_{3}+8{{\rm{H}}}_{2}{\rm{O}}.$$

The stability window for the formation of malachite in the mixed solution is therefore between pH 5.6 and 8.5. To evaluate the effect of the pH of the mixed solution on the size and shape of the copper-containing precursor phase (and thus on the final CuO end product), additional synthesis routes were performed at a fixed pH value of either 5.6 or 7.0 by adjusting the added volume of the ammonia carbonate solution, resulting in [CO_3_^2−^]/[Cu^2+^] ratios of 0.4 and 2.5, respectively. Depending on the regulated pH, either an ammonium carbonate deficiency (i.e. [CO_3_^2−^]/[Cu^2+^] < 1 for pH < 5.83) or surplus [CO_3_^2−^]/[Cu^2+^] > 1 for pH > 5.83) is established, which affects the supersaturation level of the mixed solution with respect to the nucleation of the targeted malachite precursor phase. Unless stated otherwise, the mixed solution was continuously stirred over the defined duration time of *t*_stirring_ = 2 h. Afterwards, the gel precipitate was collected by centrifugation, washed with ethanol and distilled water. The freshly washed precipitate was then dried in a muffle furnace in air at a temperature of 60 °C for a fixed duration of *t*_*drying*_ = 6 h. The resulting (largely) dehydrated precursor phase was decomposed into CuO by annealing in a muffle furnace in air at 400 °C for a fixed duration of *t*_*calcination*_ = 4 h (further referred to as calcination), i.e.

3 $${{\rm{CuCO}}}_{3}\cdot {\rm{Cu}}{({\rm{OH}})}_{2}\to 2{\rm{CuO}}+{{\rm{CO}}}_{2}+{{\rm{H}}}_{2}{\rm{O}}$$

**Figure 1:**
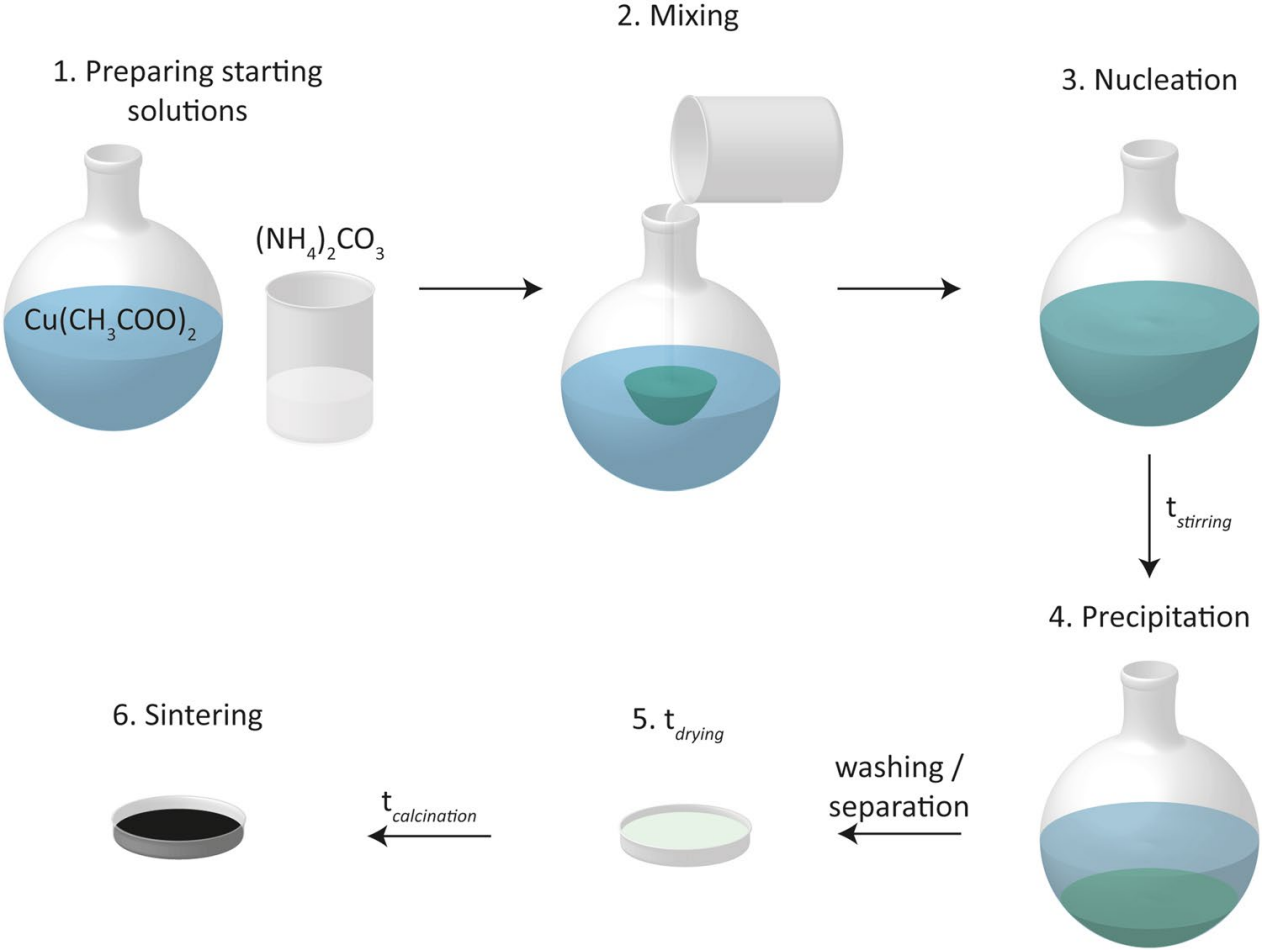
Schematic of the executed steps in the sol-gel synthesis and subsequent calcination process.

A schematic of the above-described synthesis procedure is depicted in Fig. 1.

### Material characterization

The crystallinity of the gel precursor, as well as the phase purity and average primary crystallite size of the synthesized CuO nanopowder, were investigated by X-ray diffraction (XRD) using a PANanalytical X’Pert Pro Multi-Purpose (MPD) X-ray diffractometer. The XRD data were collected in the 2Θ range of 10–100° 2Θ with a step size of 0.026° using Cu-Kα_1–2_ radiation (λ_average_ = 0.15418 nm, 45 kV and 40 mA). A time-resolved XRD study of the precursor-to-CuO transformation during calcination was conducted using a PANalytical X’Pert PRO MPD X-ray diffractometer (same measurement parameters) with a gas-tight Anton Paar XRK-900 heating chamber equipped for heating and gas feeding (5850 TR, Brooks instrument) in static air. Thermo-gravimetric analysis (TGA) combined with differential scanning calorimetry (DSC) was conducted using a Netzsch STA449 F3 Jupiter with a low-temperature silver oven. The sample (mass: ~31.6 mg) was handled in an Al sample pan (85 μl) and heated from room temperature to 400 °C at a rate of 5 K/min under flow of synthetic air (60 ml/min). The data were corrected by an empty pan scan measured under identical conditions. The morphology and elemental composition of the calcinated nanopowder was investigated using a Hitachi S-3700N scanning electron microscope (SEM), equipped with an energy-dispersive X-ray detector (EDX) (Ametek EDAX, Octane Pro). In addition, high-resolution SEM investigations were conducted using a FEI Nova NanoSEM 230. For the analysis by transmission electron microscopy (TEM; JEOL JEM-2200FS operated at accelerating voltage of 200 kV), the synthesized particles were dispersed in ethanol and transferred onto a gold grid (300 mesh, TED PELLA, INC). ATR-FTIR analysis of the different precursor samples were performed with a Cary 640 FTIR spectrometer (Agilent). The diamond ATR accessory with a type IIa synthetic diamond crystal has a penetration depth of ~2 µm. The spectra were recorded in a frequency range of 4000–600 cm^−1^ with a spectral resolution of 2 cm^−1^. A total of 128 scans were co-added for every spectrum. The background was measured with the same settings against air. The spectrometer was controlled by Agilent Resolutions Pro software 5.2.0. DLS experiments were conducted to determine the size distribution of the CuO NP aggregates. To this end, the synthesized nanopowder was dispersed in a water-based solution with 0.5% sodium dodecylsulfat (SDS) as a detergent and ultra-sonicated (UP200ST with VialTweeter, hielscher) to disrupt the weakly linked agglomerates. The DLS measurements were performed with a Zetasizer Nano ZS (Malvern Instruments Ltd., Malvern, UK) equipped with a max 4 mW He–Ne laser (emitting at 633 nm). Each measurement was performed at the non-invasive backscatter angle (NIBS) of 173° at a temperature of 25 °C and was preceded by a 30 s equilibration time. The ultrasonic treatment procedure was optimized to yield a stable minimal particle size after successive DLS measurements of the dispersion (thus ensuring near-complete disruption of the agglomerates). The specific SSA of the synthesized CuO nanopowders after calcination, as well as after ultrasonic treatment and subsequent drying in air, was determined from a 5-point N_2_ adsorption BET isotherm, as measured with a Beckman-Coulter SA3100 instrument (Switzerland). Before the BET analysis, the powder samples were dried for 2 h at 180 °C in synthetic air.

## Results and Discussion

### Transformation of the precursor phase into CuO by calcination

**Figure 2:**
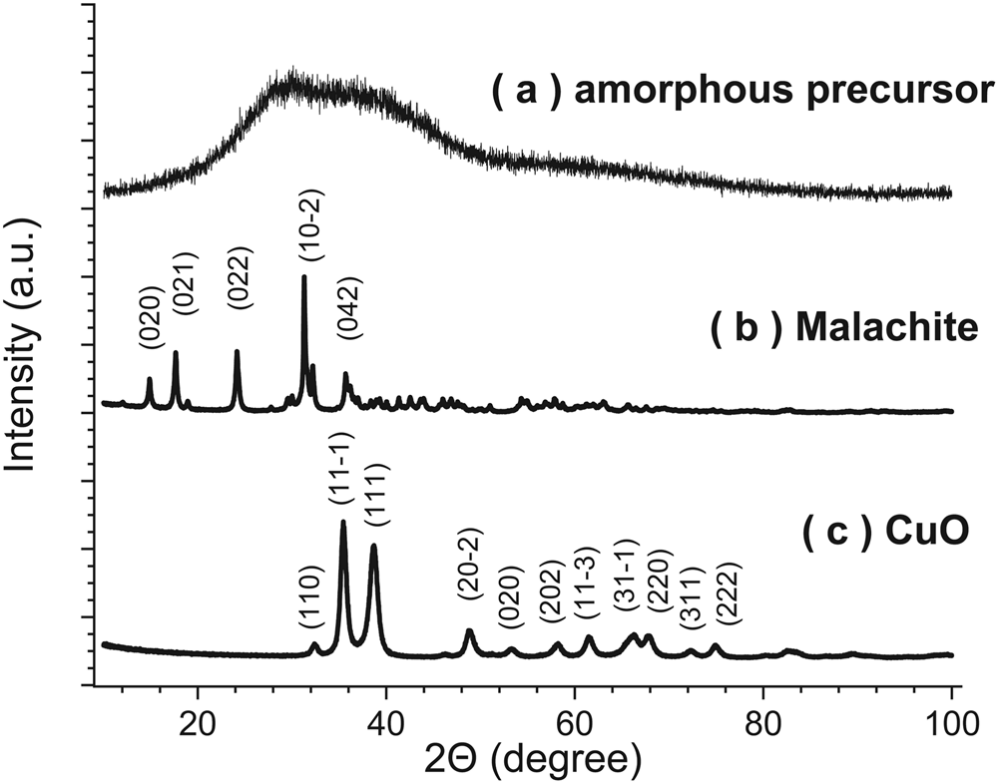
Recorded XRD diffractograms of (**a**) the freshly washed precipitate (i.e. the precursor phase) from a stoichiometric mixture, (**b**) the precursor phase after long drying at room temperature in air, which matches the reference pattern of malachite^32^ and (**c**) the final CuO product phase after calcination, which matches the reference pattern of CuO^33^.

The nature of the freshly-washed blueish-greenish precipitate collected for [CO_3_^2−^]/[Cu^2+^] = 1.0 at pH = 5.83 (step 5 → 6 in Fig. 1) to CuO was first investigated by XRD (Fig. 2). The measurement indicates that the precipitated gel is XRD-amorphous, see Fig. 2a. Only a very broad intensity hump characteristic of an amorphous phase was detected in the 2-theta region of the expected principal (10-2) reflection of crystalline malachite, but no indications of crystalline CuO were found. To confirm the presence of a malachite-like precursor phase, the obtained blue-greenish precipitate was slowly dried to completion over 48 h at room temperature (RT) in air (*note:* for the standard synthesis route, the precipitate is dried at an elevated temperature for much shorter times, as described above). The XRD pattern after drying indeed matched the crystalline structure of malachite, which crystallizes in a monoclinic space group P21/a1 with lattice parameter a = 9.502 Å, b = 11.974 Å, c = 3.24 Å, alpha = 90.00°, beta = 98.75° and gamma = 90.00°^32^: see Fig. 2b. These findings suggest that the amorphous precursor phase is chemically and structurally similar to crystalline malachite. Subsequent calcination of the thus-obtained crystalline malachite phase (as obtained by slow drying at RT in air), as well as of the amorphous precursor phase (obtained using the default synthesis route), both lead to the formation of CuO: see Fig. 2c and Eq. (3). CuO crystalizes in the monoclinic space group C12/c1 with lattice parameters a = 4.6837(5) Å, b = 3.4226(5) Å, c = 5.1288(6) Å, alpha = 90.000°, beta = 99.54(1)° and gamma = 90.000°^33^.

**Figure 3:**
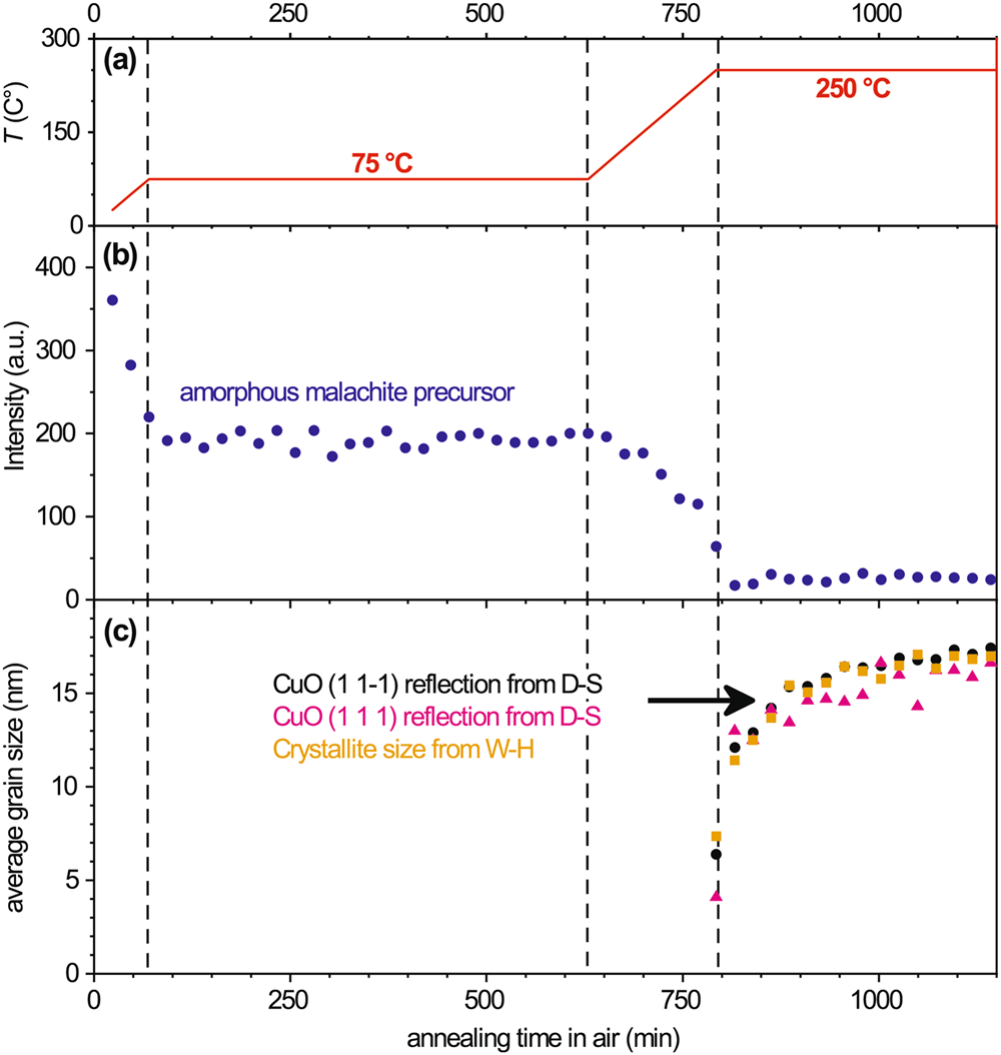
*In-situ* XRD analysis of the calcination process of the amorphous copper-carbonate-hydroxide precursor phase into CuO upon annealing in air. (**a**) Temperature profile. (**b**) Intensity of the (102) reflection over time (temperature). (**c**) Size of the coherent scattering domains of CuO as extracted from the CuO (11-1) and CuO (111) reflections using either the Debye-Scherrer or the Williamson-Hall equation.

In a next step, the calcination of the amorphous precursor gel, as obtained from the standard synthesis procedure was studied by *in-situ* HT-XRD: see Figs 3 and S1. To this end, a drying step was performed first for 12 h at 75 °C (i.e. 15 K higher than the default drying step), after which the temperature was step-wise increased with a rate of 1 °C/min to 250 °C, followed by an isothermal annealing step of 8 h at 250 °C (see Fig. 3a). In parallel, the reflections of the 2Θ region from 20–40°, which contains the principle reflections of crystalline malachite, Cu(OH)_2_, CuCO_3_ and CuO (cf. Fig. 2), were recorded by XRD. During the drying step at 75 °C, only a constant broad intensity hump, typical for the amorphous precursor phase (cf. Fig. 2a), was observed: see Fig. 3b. Upon heating to 250 °C, this diffuse intensity hump decreases, but no principle reflections of crystalline malachite were detected. Only when the temperature reached 250 °C, sharp (111) and (11-1) (and to a lesser extend (110)) reflections of CuO started to appear in the recorded diffractograms. The average size (*D*) of the developing CuO nanocrystallites during calcination at *T* = 250 °C can be estimated from the peak broadening change of the CuO(111) and CuO(11-1) reflections using the Debye-Scherrer (D-S) Eq. (4) and the Williamson-Hall formula (W-H) (5), i.e.

4 $${\rm{D}}=\frac{K\lambda }{\beta \,\cos ({\rm{\Theta }})},$$

5 $$\beta \,\cos \,{\rm{\Theta }}=4\varepsilon \,\sin \,{\rm{\Theta }}+\frac{K\times \lambda }{D},$$

where *β* is the full width at half maximum of the respective reflection at the Bragg angle 2*Θ*, *K* is a numerical factor (here: *K* = 0.9, which is a good approximation for spherical particles in the absence of detailed shape information^34–36^), ε is a factor for the strain induced peak broadening and λ is the incident X-ray wavelength (here Cu-Kα)^37,38^.

As evidenced by Fig. 3c, the average size of the CuO nanocrystallites synthesized at pH = 5.8 initially increases rapidly with increasing annealing time at *T* = 250 °C, reaching an average final size of roughly 17 ± 2 nm (by D-S) and 17.4 ± 0.4 nm (by W-H) within a few hours. Here it is emphasized that the XRD analysis probes the average size of the coherent scattering domains and therefore presents a measure of the smallest CuO crystalline building block only (and not of the CuO NP aggregate size). Notably, the Debye-Scherrer analysis on the basis of the CuO(111) and CuO(11-1) reflections gives similar crystallite sizes, which indicates that roughly equiaxed CuO crystallites are formed (*i*.*e*. single crystallites with a platelet or needle morphology are of minor importance).

After complete calcination, only reflections that match the reference pattern of CuO remain (see Fig. 2c), which suggests that the amorphous precursor phase is fully transformed into single-phase CuO within several hours of calcination at *T* ≥ 250 °C, in accordance with ref.^30^.

Differential scanning calorimetry (DSC) coupled with thermal gravimetric analysis (TGA) was carried out in synthetic air (60 mL/min) and at a heating rate of 5 K/min to 400 °C as shown in Fig. S2. An exothermic effect was detected at ~190 °C with a subsequent endothermic effect at ~260 °C, associated with a pronounced weight loss. The exothermic effect can be attributed to the crystallization of the amorphous precursor phase, as formed for the default synthesis route at pH = 5.8. The following endothermic effect is caused by the subsequent thermal decomposition of the crystallized precursor phase to CuO. The relative weight loss of 29%, as detected with TGA during the thermal decomposition, is in good agreement with the theoretical value of malachite decomposition: i.e. 1 − (2 × *M*_CuO_)/*M*_malachite_, where *M*_CuO_ and *M*_malachite_ correspond to the molar masses of CuO and malachite, respectively. This corroborates the findings from XRD that the thermal decomposition of the precursor gel into CuO (in static air) becomes thermally activated at around 250 °C. A somewhat lower transformation temperature of 180 °C was reported in ref.^39^, which might be due to a slightly different nature of the precipitate phase and/or the much longer annealing time of 48 h.

Noteworthy, DSC-TGA shows a clear crystallization peak upon heating whereas no distinct crystalline malachite reflections could be detected during heating by *in-situ* XRD (see Figs 3 and S1). This can be attributed to the overlap of the crystalline malachite peaks in XRD with the broad peak of the amorphous as-prepared gel (cf. Fig. 2), which hinders unique identification of the malachite phase as long as the amorphous gel is still present.

### Effect of pH and [CO_3_^2−^]/[Cu^2+^] -ratio on the formation of the precursor phase

**Figure 4:**
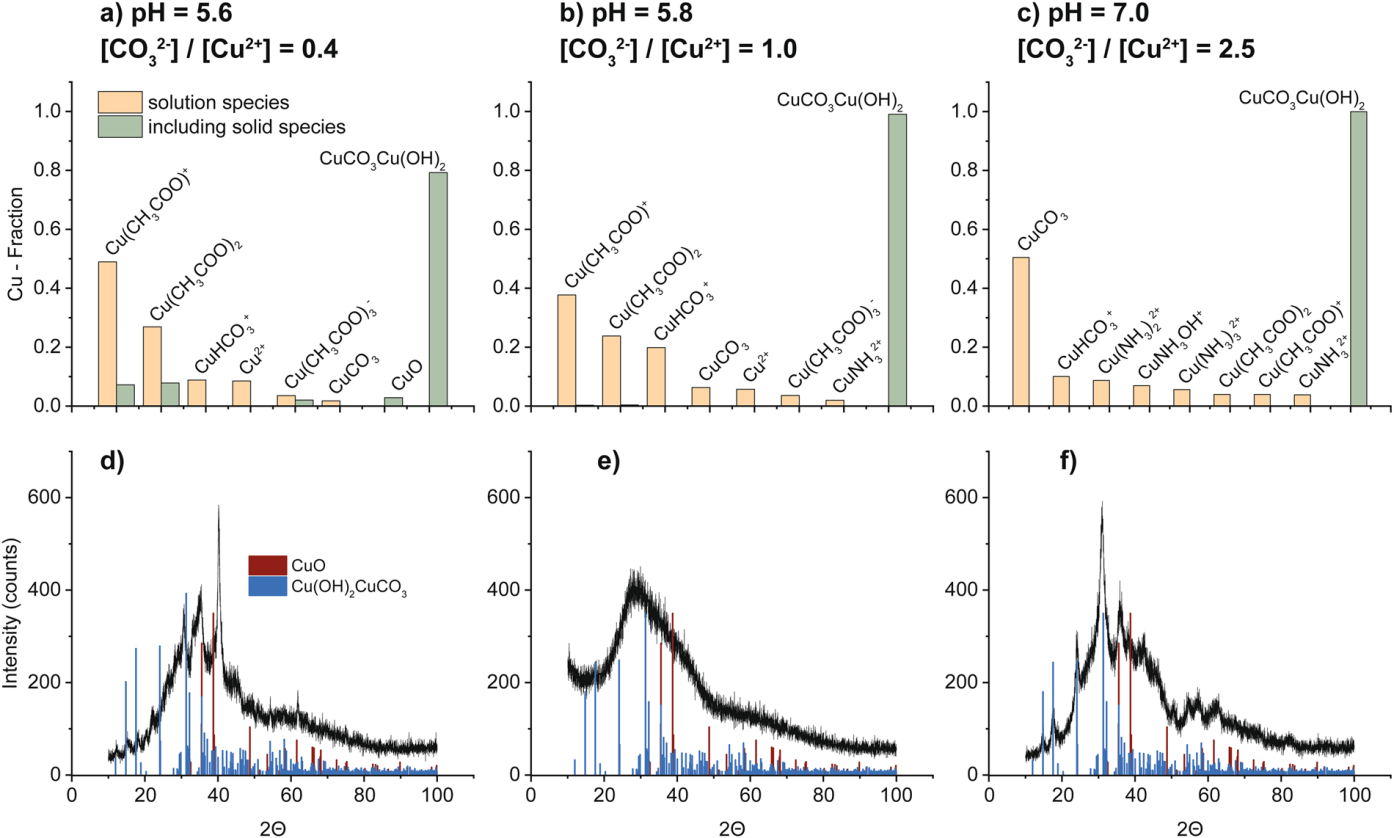
Theoretical fractions of Cu solution and precipitation species for different [CO_3_^2−^]/[Cu^2+^] concentration ratios and pH’s of the mixed solution, as calculated including (*green*) and excluding precipitation species (*orange*). Predictions for (**a**) [CO_3_^2−^]/[Cu^2+^] = 0.4 and pH = 5.6, (**b**) [CO_3_^2−^]/[Cu^2+^] = 1.0 and pH = 5.8 and (**c**) [CO_3_^2−^]/[Cu^2+^] = 2.5 and pH = 7.0. The respective XRD patterns of the experimentally collected precipitates after 2 h of stirring pertaining to the [CO_3_^2−^]/[Cu^2+^] concentration ratios and pH’s of (**a**–**c**) are shown in (**d**–**f**) respectively including reference pattern for CuO (red)^33^ and malachite (blue)^32^.

To evaluate the influence of different pH-values and [CO_3_^2−^]/[Cu^2+^]-ratios on the synthesis reaction, the mixed solution was regulated to a specific pH value of either 5.6 or 7.0 by adjusting the added volume of ammonia carbonate solution, which corresponds to an ammonium carbonate deficiency (i.e. [CO_3_^2−^]/[Cu^2+^] < 1 for pH < 5.83) or surplus (i.e. [CO_3_^2−^]/[Cu^2+^] > 1 for pH > 5.83), respectively. To provide a better insight into the precursor composition, the chemical speciation of Cu in aqueous solution, as well as the thermodynamic equilibrium with possible solid precipitate species (i.e. malachite, azurite, copper carbonate, copper hydroxide and copper oxide), were assessed as a function of pH for different carbonate concentrations using the MEDUSA software and the respective equilibrium and solubility constants from the HYDRA database^40^. The predictions were performed for various ammonia carbonate and copper acetate starting concentrations, as used in the experiments. The calculation results are plotted in Fig. 4a–c for the experimentally studied [CO_3_^2−^]/[Cu^2+^] ratios (and corresponding pH’s) of [CO_3_^2−^]/[Cu^2+^] = 0.4 (pH = 5.6), [CO_3_^2−^]/[Cu^2+^] = 1.0 (pH = 5.8) and [CO_3_^2−^]/[Cu^2+^] = 2.5 (pH = 7.0), respectively. Since the solubility of the precipitating species is relatively low, they dominate the calculation results (see green colored bars in Fig. 4a–c,). Therefore, a second set of calculations was performed, which only considered the species remaining in solution (orange colored bars in Fig. 4a–c). The respective XRD patterns which were experimentally collected for the precipitate gels after 2 h of stirring are plotted for comparison in Fig. 4d–f.

For a carbonate deficiency of the mixed solution (Fig. 4a, pH = 5.6), [Cu^2+^∙(OAc)^−^]^+^, [Cu^2+^∙2(OAc)^−^] (with OAc = CH_3_COO) and [Cu^2+^∙(HCO_3_)^−^]^+^ are the dominant Cu complexes in solution. Due to the low solubility of malachite, the equilibrium is largely shifted towards the solid malachite phase (compare calculation with and without precipitation species in Fig. 4). According to the thermodynamic equilibrium assessment, Cu can to some extend also precipitate as CuO. Indeed, as predicted both malachite and CuO are being detected by XRD in the (freshly washed) precipitated gel, as collected after the default stirring duration of 2 h (see step 4 → 5 in Fig. 1). The CuO signal appears to be much more dominant than that of malachite in the recorded diffractograms, which would implicate that the formation of malachite is suppressed compared to CuO under carbonate-deficient conditions, in contrasts to the thermodynamic assessment. However, the crystalline reflections of malachite and CuO are superimposed on a very broad intensity hump indicating the presence of an amorphous (malachite-like) precipitate phase. In this case, the summed-up signal intensity from crystalline malachite plus the amorphous malachite-like precursor clearly dominates over that of CuO, in accordance with the model predictions. For the [CO_3_^2−^]/[Cu^2+^] ratio equal to 1.0, which corresponds to a slightly higher pH of 5.8, similar calculation results are obtained, although the CuCO_3_-complexes get slightly more dominant and the equilibrium further shifts to malachite, being by far the most dominant precipitating species (*note:* the predicted fraction of CuO becomes negligible, Fig. 4b). The freshly washed precipitate collected for the 1:1 ratio mixture is fully XRD-amorphous (Fig. 4e), but transforms into crystalline upon complete drying in air (see Fig. 2b). Finally, the calculations for a carbonate surplus of [CO_3_^2−^]/[Cu^2+^] = 2.5 (pH = 7.0) predict CuCO_3_ as the most dominant solution species and malachite as the only precipitation species; the acetate-containing copper complexes have only a minor contribution and the [Cu^2+^∙(HCO_3_)^−^]^+^ contribution is increased (Fig. 4c). In this case, the XRD analysis of the freshly-washed precipitate indeed only shows crystalline malachite, as well as the amorphous copper-carbonate-hydroxide (malachite-like) precursor phase (Fig. 4f).

The three different wet precursors with [CO_3_^2−^]/[Cu^2+^] = 0.4, 1.0 and 2.5 were also analyzed by ATR-FT-IR to disclose the differences in their molecular compositions. The recorded ATR-FT-IR spectra are shown Fig. S3 in the Supplementary Information. In all cases, vibration bands at 3311 cm^−1^, 2400–1900 cm^−1^ and 1637 cm^−1^, corresponding to vibrations of the H_2_O solvent were identified. The FTIR spectra do not show any characteristic vibration bands for copper acetate^41^ or acetate ions^42^, which indicates that acetate is not contributing to the precursor phase and is removed during the washing process. The vibration detected at 880 cm^−1^ for [CO_3_^2−^]/[Cu^2+^] = 1.0 and 2.5 corresponds to the non-planar rocking of bonded CO_3_^2−^, which does not appear for the sample with carbonate deficiency (i.e. [CO_3_^2−^]/[Cu^2+^] = 0.4)^43^. Also the absorption bands for the symmetric C-O stretching at 1045 cm^−1^ and 1085 cm^−1^ appear less intense for the sample with carbonate deficiency. The largest differences in FTIR spectra for the three synthesis conditions are detected in the region of 1300–1530 cm^−1^, in which asymmetric C-O stretching and CO_2_ stretching vibrations of carbonate species appear^43^. The precursor obtained from the carbonate surplus reaction shows an absorption at ~1510 cm^−1^, corresponding to asymmetric C-O stretching in basic conditions of complexated carbonates (fully deprotonated CO_3_^2−^)^43^ and at ~1400 cm^−1^ (CO_2_ stretching in carbonates). Its small shoulder at 1420 cm^−1^ coincides with the broad absorption of the [CO_3_^2−^]/[Cu^2+^] = 0.4 sample. In refs^43^ and^44^ it was shown that the carbonate vibrations in acidic environments and in bicarbonate compounds shift as compared to their carbonato counterparts. The asymmetric C-O stretching vibrations in acidic environments shift towards 1620–1660 cm^−1^ and are thus covered by the absorption band of the solvent. The CO_2_ stretching vibrations in HCO_3_^−^ compounds appear at ~1410 cm^−1^ and ~1475 cm^−1^. Hence the FTIR analysis reflects a dominant contribution of HCO_3_^−^ species (1410 cm^−1^ and 1475 cm^−1^) and only a minor contribution of CO_3_^2−^ species (visible at 1045 cm^−1^) at a carbonate deficiency, in agreement with the theoretical assessment of the solution species. Accordingly, the CO_3_^2−^ vibrations (1510 cm^−1^ and 1400 cm^−1^) in basic/neutral environments are dominant in the sample prepared with [CO_3_^2−^]/[Cu^2+^] = 2.5. The precursor obtained from a starting solution of [CO_3_^2−^]/[Cu^2+^] = 1.0 shows no dominant carbonate vibrations in the 1300–1530 cm^−1^ region. Weaker absorption bands that coincide with the absorption bands for CO_3_^2−^ and HCO_3_^−^, as expected for [CO_3_^2−^]/[Cu^2+^] = 2.5 and 0.4, can be identified. The FTIR analysis thus indicates that the amorphous precursor phase, as formed for the default synthesis route at pH = 5.8, is predominantly constituted of copper carbonate and bicarbonate species and can thus be designated as an “*amorphous malachite-like precursor phase*”. In conclusion, the experimental findings in combination with the thermodynamic calculations indicate that the observed amorphous phase is composed of randomly-packed clusters and chains of the predicted copper-complexes with HCO_3_^−^, CO_3_^2−^ and H_2_O ligands (see Fig. 4). For the stoichiometric ratio of [CO_3_^2−^]/[Cu^2+^] = 1.0, this amorphous precursor phase is the dominant product phase of the complex forming reaction and preferably convert into *crystalline* malachite upon slow drying and/or subsequent calcination. For deviations from the ideal ratio (and its corresponding pH-value), the crystallization of malachite from the amorphous precursor phase seems to be accelerated, but also the crystallization of CuO can then be observed.

### Effect of pH, [CO_3_^2−^]/[Cu^2+^] -ratio and precipitation time on the morphology and size of the calcined CuO nanopowder

In order to tailor the crystallite and agglomerate sizes of the CuO nanopowder, the influence of the solution concentrations and resulting pH on the size and shape of the synthesized CuO NP aggregates as function of precipitation time was investigated in more detail. Since the hierarchical structures of the Cu precursor phase are generally largely preserved upon thermal decomposition into CuO by calcination^22^, the effect of the calcination treatment on the synthesized CuO product was not specially considered in the present study. Therefore, all calcinations were performed for 4 h at 400 °C.

**Figure 5:**
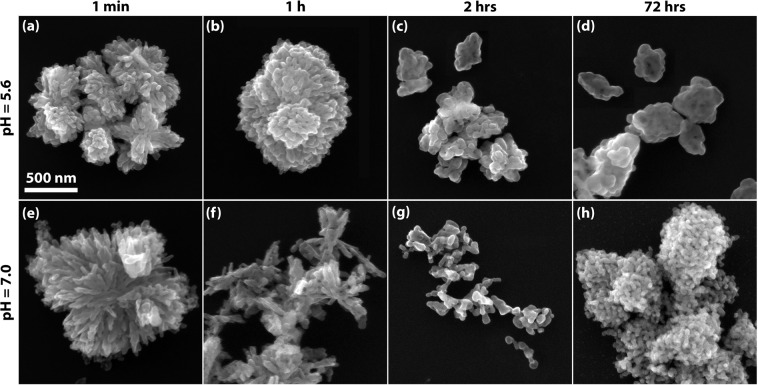
SEM images of CuO nanoparticles synthesized at different pH-values and precipitation (i.e. aging) times of the solution. The aging time of 2 h corresponds to the optimum aging time.

Calcination of the precipitate collected after different stirring times of 1 min, 1 h, 2 h and 72 h leads to a clear difference in shape and compactness of the primary CuO crystallites and aggregates for the two [CO_3_^2−^]/[Cu^2+^]-ratios considered, as becomes apparent from the SEM analysis of the CuO nanopowder (Fig. 5). For a very short stirring time of 1 min, the aggregated NPs and agglomerates produced at both pH values are predominantly constituted of large crystallites with needle- and platelet-like morphologies. This anisotropic crystallite shape found at the onset of mixing hints at the presence of an intermediate precursor phase which differs from both the above discussed copper-containing amorphous malachite-like precursor phase and crystalline malachite, which can be found after 2 h of stirring. A likely explanation is the formation of a precipitating phase similar to the amorphous malachite-like precursor phase, but with an increased hydroxide content, which is typical for the formation of needle- and platelet-shaped Cu-hydroxides^30,45^. Indeed, it may be assumed that the complexation of Cu^2+^ by (larger and less mobile) carbonate CO_3_^2−^ and bicarbonate HCO_3_^−^ ions, as associated with the nucleation and growth of a malachite precursor phase, is relatively sluggish as compared to complexation of Cu^2+^ by OH^−^. Upon further stirring, this intermediate precursor phase gradually dissolves again, as the more sluggish complexation of Cu^2+^ by the larger carbonate CO_3_^2−^ and bicarbonate HCO_3_^−^ ions progresses, leading to the formation of the amorphous malachite-like precursor phase. As follows from the structural analysis of malachite in ref. ^24^, malachite itself shows not only selective bonding along [001] crystallographic directions by the interconnection of Cu(OH)_2_ building blocks, but also produces strong Cu-O with the carbonate ions in the (001) planes^24^. Assuming a similar local ordering for the amorphous malachite-like precursor, the progressive complexation of Cu^2+^ by carbonate CO_3_^2−^ ions^46^ then results in the observed change in the crystallite shape of the CuO NPs from anisotropic to equiaxed morphology with increasing stirring time: see Fig. 5. As follows from a comparison of Fig. 5c with 5g, the (agglomerated) CuO aggregates synthesized for stirring times of 2 h appear much less densely clustered for pH = 7.0 as compared to pH = 5.6. For longer stirring times, the degree of NP aggregation increases for both pH-values, hinting at increasing densification of the amorphous precursor phase. Hence, a stirring time of 2 h can be considered as the optimum aging time for production of nano-porous nanopowders.

**Figure 6:**
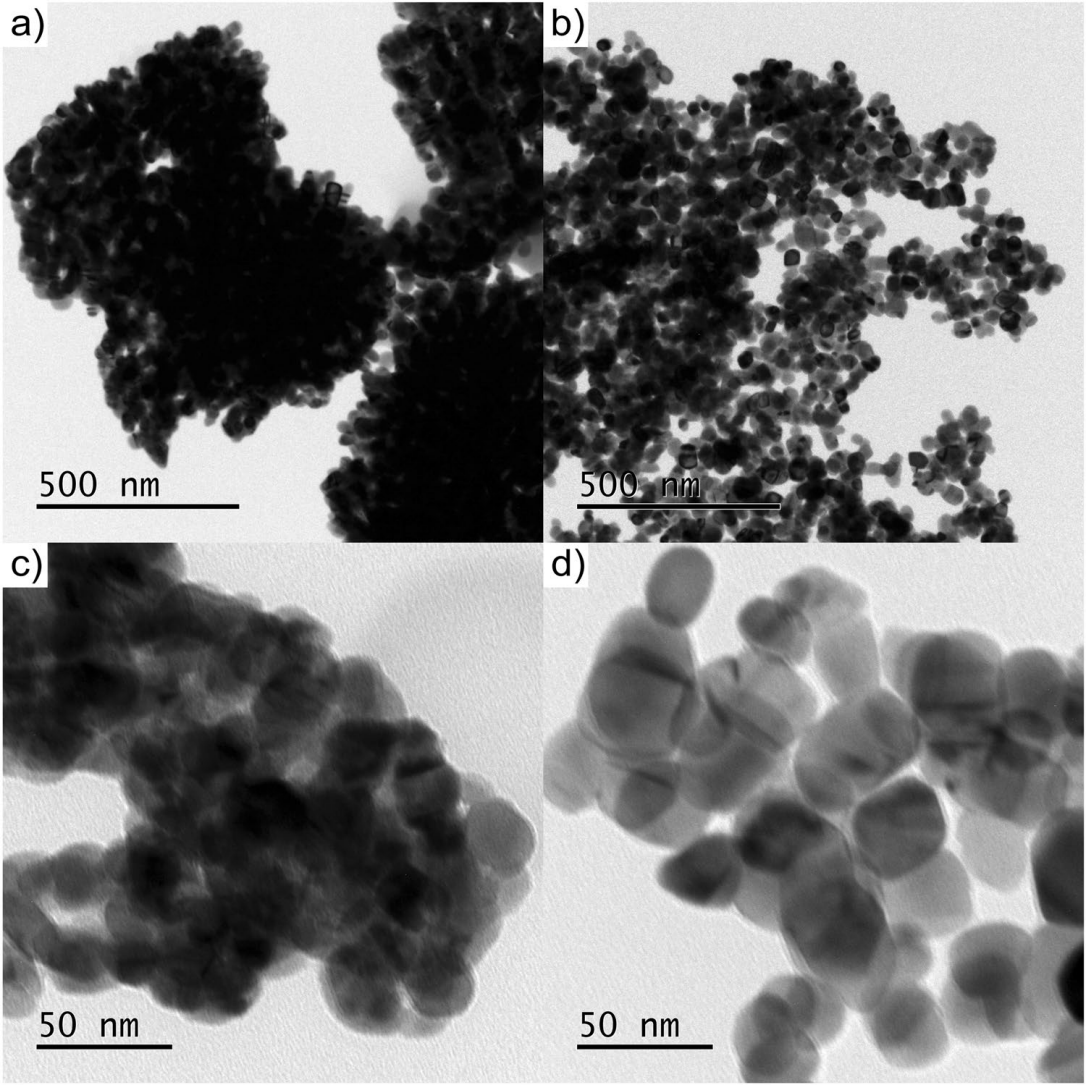
TEM images of the agglomerated CuO aggregates, as obtained after synthesis at (**a**,**c**) pH = 5.6 and (**b**,**d**) pH = 7.0. Except for the pH, all other synthesis parameters were taken as described in the material synthesis section.

The TEM micrographs of the calcinated CuO nanopowders obtained at pH = 5.6 and 7.0 for the optimum stirring time of 2 h are shown in Fig. 6. Deposition of the nanopowder dispersions on the electron-transparent TEM grid leads to a mixture of CuO aggregates and their agglomerates, which hinders a determination of the true CuO NP aggregate size (which is obtained by DSL in the present study). However, the TEM analysis clearly evidences that the agglomerated aggregates are constituted of clusters of much smaller primary crystallites. As is evident from comparison of Fig. 6c,d, the average size of the primary crystallites increases with increasing pH. The primary crystallite size of 23 ± 6 nm for pH = 5.6, as determined by TEM (see Fig. 6a), complies well with the CuO crystallite sizes of 20 ± 6 nm (from D-S) and 23 ± 9 nm (from W-H), as determined by XRD. Notably similar crystallite sizes were obtained in the *in-situ* HT XRD study of the calcination process (for pH = 5.8) as discussed above. For pH = 5.6, CuO particles with considerably larger crystallite and aggregate sizes could be observed by TEM in very few occasions. These much larger particles may be attributed to the observed formation of fewer primary CuO crystallites during the synthesis step. Investigation of the obtained particles from pH = 7.0 show a larger average crystallite size of 38 ± 8 nm which also complies reasonably well with the corresponding XRD values of 25 ± 7 nm (for D-S) and 28 ± 13 nm (for W-H). Notably, for the synthesis at pH = 7.0 the shape of the primary crystallites appears smoother and more spherical, and the resulting (agglomerated) CuO aggregates are more loosely packed, corroborating the observations made by SEM: compare Fig. 6a–d.

A possible explanation for the observed difference in primary crystalline as well as in aggregate size and morphology between pH = 5.6 and pH = 7.0 is the following. For the pH = 5.6 solution, a [CO_3_^2−^]/[Cu^2+^] = 0.4 ratio induces a deficiency of CO_3_^2−^ ions with respect to Cu^2+^ ions (see FTIR analysis). It may be assumed that upon gel formation, i.e. condensation of the Cu-complexions towards the nominal malachite composition, a much denser network of Cu^2+^ and CO_3_^2−^ is developed than in case of an equimolar ratio of Cu^2+^ and CO_3_^2−^, i.e. more Cu-carbonate-Cu-bridges are formed. As a consequence the formed gel is much more compact, and calcination of the gel then leads to larger primary CuO crystallites with higher cluster density, i.e. denser aggregate morphology.

The observed difference in primary crystalline and aggregate size between pH = 5.6 and pH = 7.0 can be rationalized as follows: upon mixing of the starting solutions, the concentrations of the competing Cu complexation species practically instantaneous reach critical supersaturation, which for the ideal case of a homogeneous mixed solution under thermodynamic equilibrium, would result in the instantaneous homogeneous nucleation of the most stable precursor phase (i.e. crystalline malachite). These initial nuclei can grow upon aging of the mixed solution by a combination of diffusion-limited growth and aggregation (i.e., by Ostwald ripening through dissolution, growth and impingement of existing nuclei)^47^. The mixed solution at pH = 5.6 has a much lower [CO_3_^2−^]/[Cu^2+^]-ratio and thus a much lower degree of supersaturation (see Eq. [1]). Moreover, as previously discussed, Cu complexes with HCO_3_^−^ and (OAc)^−^ ligands dominate at [CO_3_^2−^]/[Cu^2+^] = 0.4 and pH = 5.6, whereas [CuCO_3_] complexes become more dominant at [CO_3_^2−^]/[Cu^2+^] = 2.5 and pH = 7.0 (compare Fig. 4a,c). The CuO aggregates (as obtained after calcination), as synthesized from a carbonate deficient solution at pH = 5.6, are much more compact than the ones synthesized in a carbonate enriched solution at pH = 7.0 (compare Fig. 6d,f, as well as Fig. 5a,c,d). Here we propose the following explanation for the much higher compactness of the CuO aggregates synthesized at pH = 5.6. The precipitating amorphous precursor phase consists of randomly-packed clusters and chains of the predicted copper-complexes with HCO_3_^−^ and CO_3_^2−^ ligands (see Fig. 4), resulting in an amorphous precursor phase. The lower the [CO_3_^2−^]/[Cu^2+^]-ratio, the closer the proximity between neighboring copper cores in the precipitated amorphous precursor gel, which upon calcination result in a more dense CuO aggregates. Finally it is noted that, according to LaMer’s-Model, larger primary crystallites are formed at a lower supersaturation (i.e. pH = 5.6), which is not observed in this study: the primary crystallites formed at pH = 5.6 are smaller than those grown at pH = 7.0 (as evidenced by XRD and TEM). In this regard it is emphasized that a variation of the carbonate molarity not only affects the supersaturation, but also the pH of the solution and thereby the stability (i.e. solubility) of the solid precursor phase in the mixed solution as well as its composition (thus affecting its final size resulting from Oswald ripening during continuous stirring). Both the higher carbonate molarity and the higher stability of the malachite phase promote diffusion-limited growth of the primary malachite nuclei at pH = 7.0.

### Determination of the CuO aggregate size by DLS

**Figure 7:**
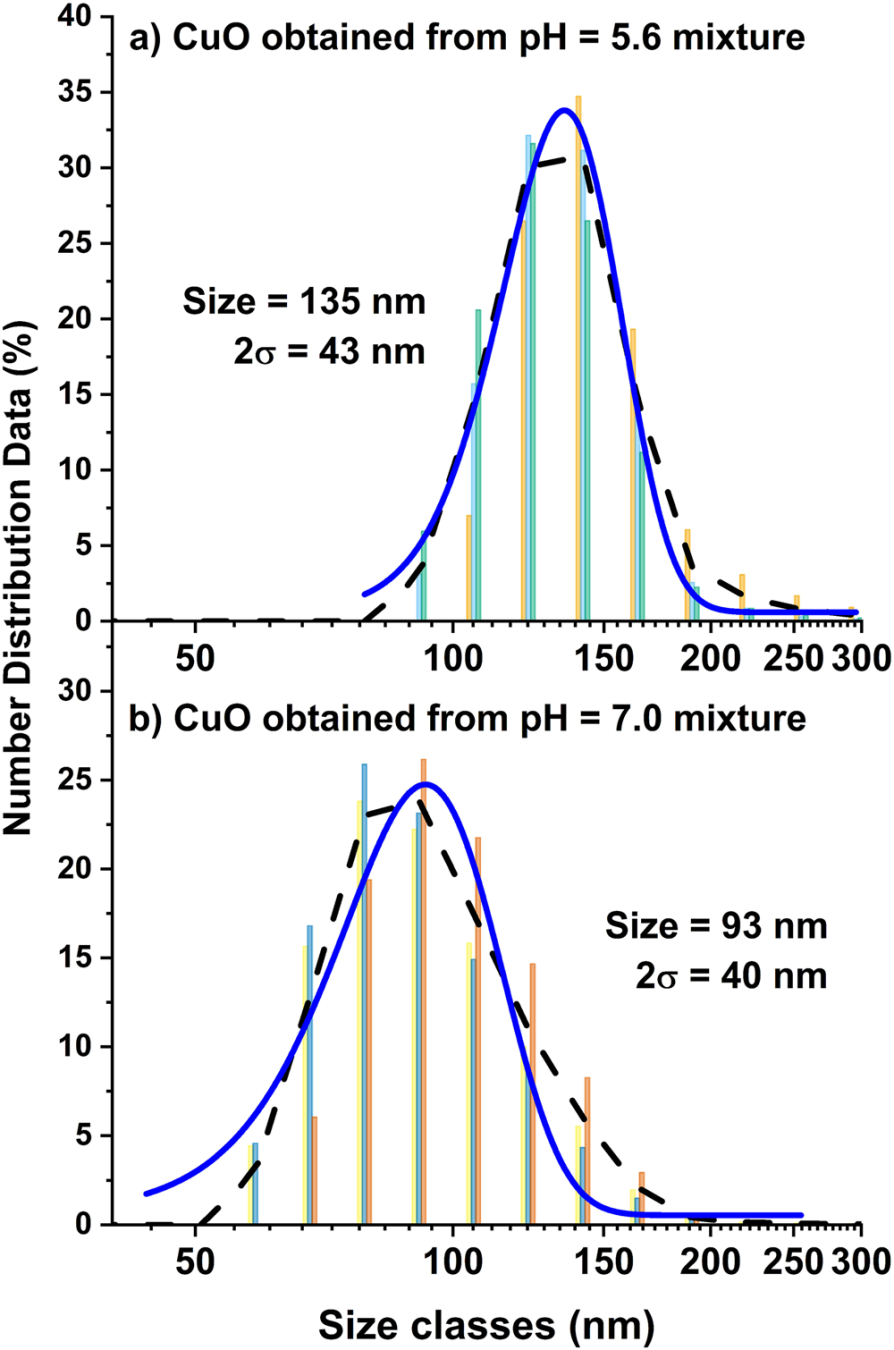
Size distributions of CuO aggregates synthesized at (**a**) at pH = 5.6 and (**b**) pH = 7.0, as measured by DLS. The synthesized CuO nanopowders were dispersed in a water-based solution with 0.5% sodium dodecyl sulfate (SDS) using ultrasound waves and subsequently measured by DLS. Colored bars indicate single measurement; black line is the average of these measurements and blue line is the Gaussian fit of the average values.

DLS was applied to determine the average size of the CuO aggregates, as dispersed in a water-based solution with 0.5% sodium dodecyl sulfate (SDS) following an ultrasound treatment. In this regard, it is emphasized that DLS records the intensity of the scattered light at very high temporal resolution from which the hydrodynamic diameter is calculated, which is the diameter of the particle or aggregate plus any ligands, ions or molecules that are attached to their surface (*here:* SDS). The ultrasonic treatment procedure was optimized to yield a stable minimal particle size after successive DLS measurements of the same solution. Typical measurements of the number distribution (i.e. the number of particles in different size bins) of the CuO nanopowders synthesized at pH = 5.6 and 7.0 are plotted in Fig. 7a,b, respectively.

The DLS analysis gives an average CuO aggregate size of 135 nm ± 43 nm (PDI = 0.301) at pH = 5.6 and of 93 nm ± 40 nm (PDI = 0.167) at pH = 7.0 based on the analysis of the number size distribution. Hence the compact CuO aggregates formed at pH = 5.6 are, on average, larger than the loosely-clustered aggregates synthesized at pH = 7.0. Notably, both aggregate sizes are in the sub-micron range, as required for the targeted applications.

### Determination of the specific surface area (SSA) of the CuO nanopowder by BET analysis

The specific surface area (SSAs) of the synthesized CuO nanopowders after calcination, as well as after ultrasonic treatment and subsequent drying in air, were determined by BET analysis. The compact CuO aggregates synthesized at pH = 5.6 have an SSA of 16 m²/g, which can be compared with typical SSA values in the range of 10–15 m²/g, as indicated for commercially available high-purity (i.e. ≥99.99%) CuO nanopowders (e.g. US research Nanomaterials^©^, Plasmachem^©^, GetNanoMaterials^©^, Nanografi^©^ and Nanoshel^©^). In a next step, the same CuO nanopowder (i.e. synthesized at pH = 5.6) was dispersed in a water-based solution with 0.5% SDS using ultrasound waves and dried in air. Strikingly, a similar SSA of 15.33 m²/g was determined after the ultrasonic treatment and subsequent drying. This indicates that the ultrasonic treatment of the nanopowder dispersion is not affecting the effective surface area. In other words, although the more weakly linked agglomerates will be dispersed during the ultrasonic treatment, this has no distinct effect on the effective available surface area of compact aggregates.

The loosely-clustered CuO aggregates synthesized at pH = 7.0 (with an average size of 93 nm; see Fig. 7b) have an SSA of 18.73 m²/g before and 18.92 m²/g after the ultrasonic treatment, which is about 20% larger than the SSA of the compact CuO aggregates (with an average size of 135 nm; see Fig. 7a).

**Figure 8:**
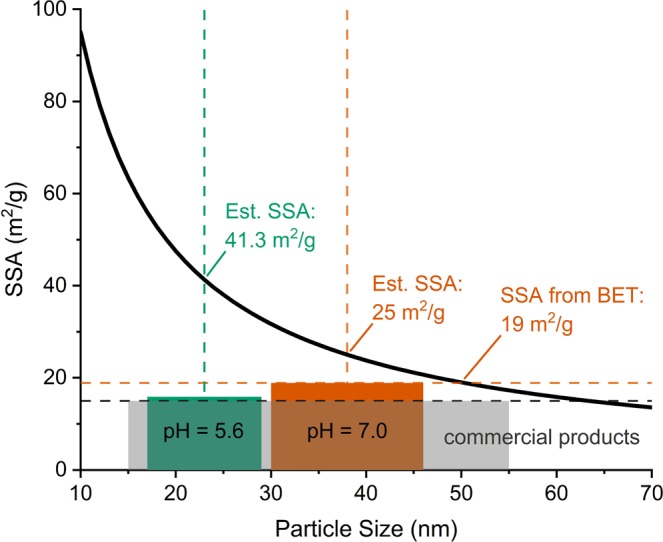
Calculated theoretical SSA for CuO particles of different diameters and experimental results obtained in the present study for different synthesis conditions. Indicated are the theoretical SSAs for particles obtained from the measured crystallite size as well as the SSAs measured with BET. For comparison the SSA indicated for typical commercial products is shown.

The maximum theoretical SSA that can be achieved for spherical nanoparticles is plotted as function of the particle diameter in Fig. 8. The SSA’s of the compact and nanoporous CuO aggregates, as synthesized in this study are compared with the indicated SSAs for commercially available CuO nanopowders. It follows that the SSA of ≈19 m²/g, as obtained for the nanoporous CuO NP aggregates formed from pH 7 solution in the present study, is not only significantly higher than for most commercial CuO nanopowders, but also much closer to the theoretical value of ≈25 m²/g for the respective primary crystallite size (by TEM) of 38 ± 8 nm. Strikingly, the SSA for the compact CuO aggregates with a smaller primary crystallite size of 23 ± 6 nm, as synthesized at pH = 5.6, falls far below the respective theoretical SSA. Although a smaller primary crystallite size should result in a higher SSA (see Fig. 8), this was not observed in the present study. It is thus concluded that the BET analysis effectively probes the enhanced nanoporosity of the loosely-clustered CuO aggregates synthesized at pH = 7.0: i.e. the nanoporosity of the CuO aggregates at pH = 7.0 is much better accessible (permeable) to the infiltration by a gas (or liquid) than in case of the denser aggregates for pH = 5.6.

These findings underline the importance of tuning the cluster density of the primary crystallites in stable NP aggregates (and not solely the primary crystallite size) for achieving a high SSA and thereby an enhanced chemical reactivity (often the main property aimed at with the use of nanoparticles) and sinterability of the nanopowder when processed in the form of a dispersed solution, paste or nanocomposite. First trials of low temperature joining with the less dense CuO NP aggregates processed as a nanopaste have confirmed that the sintering temperature and time for the bonding process can both be effectively lower by tuning the SSA of the CuO NP aggregates (work in progress).

## Conclusion

The cost-effective and green sol-gel synthesis of CuO nanopowders via thermal decomposition of an amorphous malachite-like precursor phase was successfully implemented to tune size, shape and cluster density of the primary crystallites in the CuO NP aggregates. *In-situ* heating XRD measurements showed that the transformation of the amorphous malachite-like precursor phase into single-phase CuO upon heating in air becomes thermally activated at *T* ≥ 250 °C. The resulting CuO nanopowder is composed of (agglomerated) CuO aggregates, which are constituted of clusters of much smaller primary CuO crystallites. The size, shape and nanoporosity (i.e. aggregate cluster density) of these primary CuO crystallites can be tuned by controlled adjustment of the pH and the degree of supersaturation of the mixed solution with respect to the nucleation of malachite. To this end, the mixed solution was regulated to a specific pH value of either 5.6 or 7.0 by adjusting the added volume of ammonia carbonate solution, which resulted in an carbonate deficiency (i.e. [CO_3_^2−^]/[Cu^2+^] < 1 for pH < 5.83) or surplus (i.e. [CO_3_^2−^]/[Cu^2+^] > 1 for pH > 5.83), respectively. This resulted in an average CuO aggregate size of 135 nm ± 43 nm at pH = 5.6 and of 93 nm ± 40 nm at pH = 7.0. The loosely clustered (and thus nanoporous) CuO aggregates synthesized at pH = 7.0 have a specific surface area of 18.73 m²/g, which is about 20% larger than the SSA of 15.97 m²/g for the compact CuO aggregates, despite a smaller NP crystallite size of the latter structure. It follows that the cluster density of the primary crystallites in the synthesized CuO NP aggregates can be tailored to enhance the specific surface area of the resulting CuO nanopowder for targeted applications in the field of e.g. catalysis, nanojoining, energy conversion and energy storage.

## Supplementary information

Supplementary information

## Data Availability

The datasets generated and analysed during the current study are available from the corresponding author upon reasonable request.
