# Supplementary material for: Cost-effective sol-gel synthesis of porous CuO nanoparticle aggregates with tunable specific surface area
Source: Sci Rep. 2019 Aug 13;9:11758. doi: 10.1038/s41598-019-48020-8 (PMC6692347; doi:10.1038/s41598-019-48020-8)
Supplement: Supplementary file 1 — Supplementary information [file 41598_2019_48020_MOESM1_ESM.pdf]

Supplementary Material

Cost-effective sol-gel synthesis of porous CuO nano-particle aggregates with tunable specific surface area

Lars Dörner[1,2], Claudia Cancellieri[1], Bastian Rheingans[1], Marc Walter[2,4], Ralf Kägi[3], Patrik Schmutz[1], Maksym V. Kovalenko[2,4], Lars P.H. Jeurgens[1,*]

[1] Empa, Swiss Federal Laboratories for Materials Science and Technology, Laboratory for Joining Technologies & Corrosion, Dübendorf, Switzerland

[2] ETH Zürich, Department of Chemistry and Applied Biosciences, Zürich, Switzerland

[3] Eawag, Swiss Federal Institute of Aquatic Science and Technology, Department Process Engineering, Dübendorf, Switzerland

[4] Laboratory for Thin Films and Photovoltaics, Empa – Swiss Federal Laboratories for Materials Science and Technology, Dübendorf, Switzerland

***Correspondence to lars.jeurgens@empa.ch**

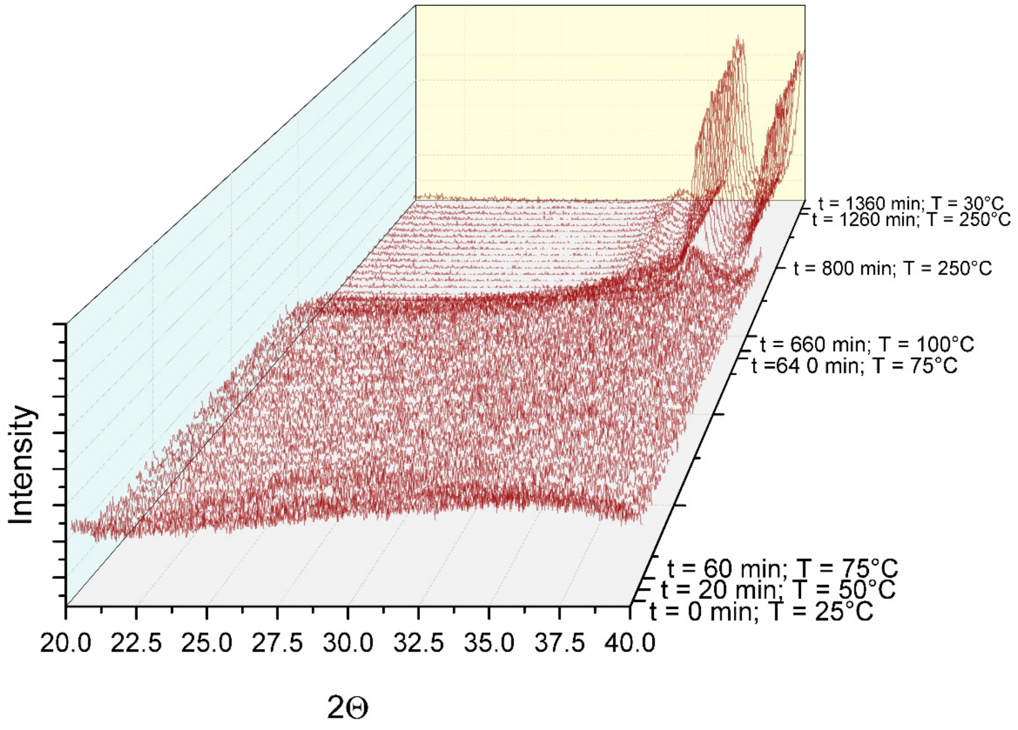

Figure S1 In-situ XRD patterns of the calcination process of the amorphous copper-carbonate-hydroxide precursor phase into CuO upon annealing in air including time and temperature steps.

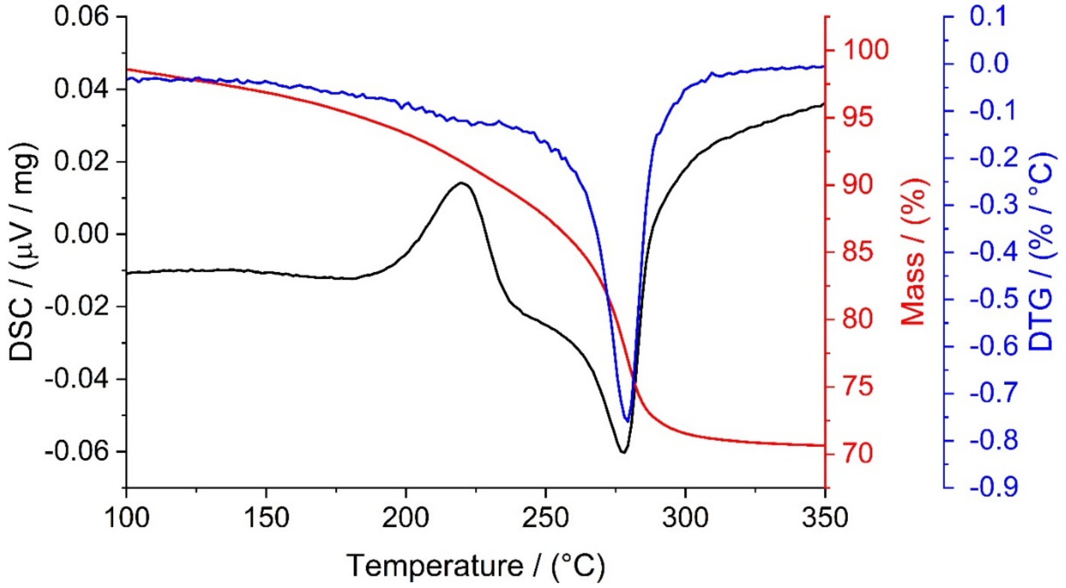

Figure S2 Differential scanning calorimetry coupled with TGA of the thermal decomposition and calcination of the amorphous copper-carbonate-hydroxide precursor phase into CuO. The mass loss and the time differential mass loss are included in this graphic.

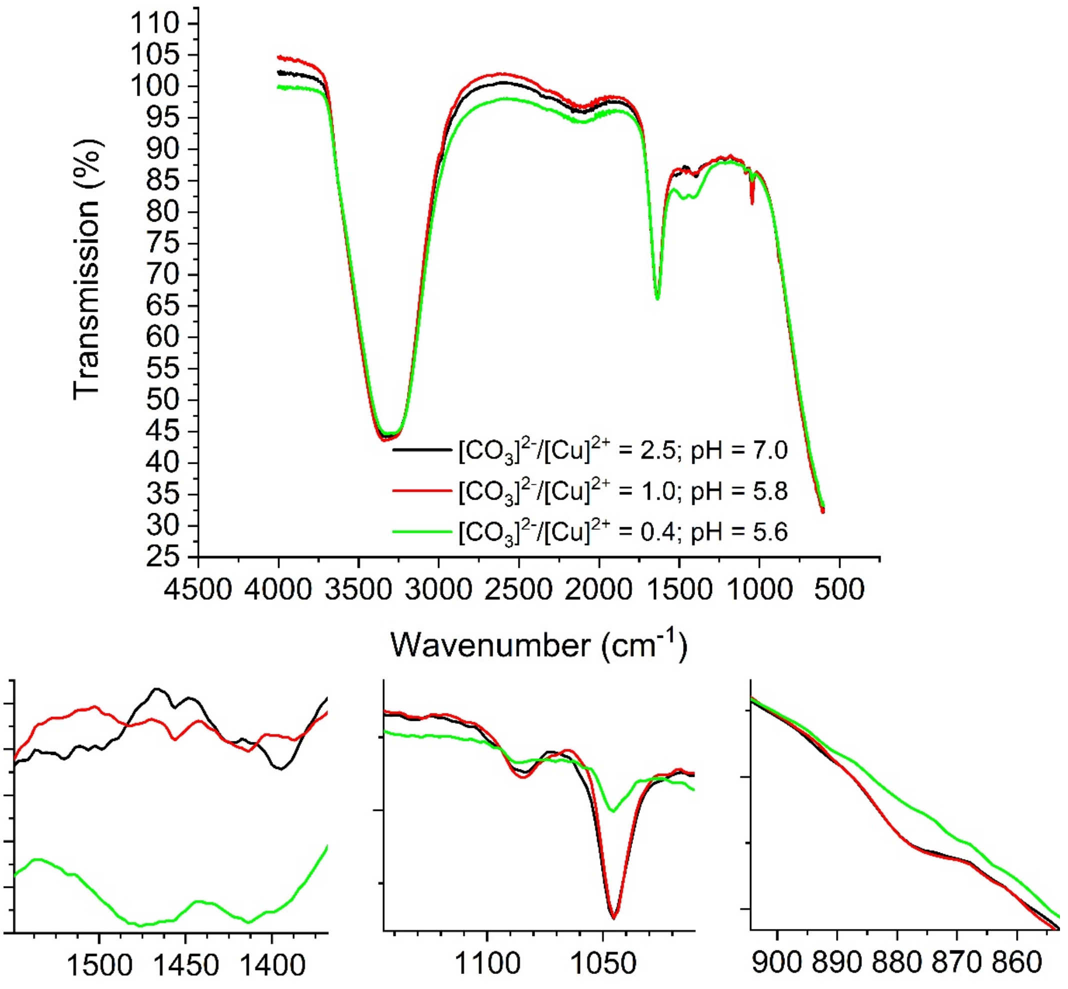

Figure S3 ATR-FT-IR spectra of the precursor prepared under different conditions.
